# Depression, Antidepressant Use and Mortality in Later Life: The Health in Men Study

**DOI:** 10.1371/journal.pone.0011266

**Published:** 2010-06-23

**Authors:** Osvaldo P. Almeida, Helman Alfonso, Graeme J. Hankey, Leon Flicker

**Affiliations:** 1 Western Australia Centre for Health & Ageing, Centre for Medical Research, University of Western Australia, Perth, Western Australia, Australia; 2 School of Psychiatry and Clinical Neurosciences, University of Western Australia, Perth, Western Australia, Australia; 3 Department of Psychiatry, Royal Perth Hospital, Perth, Western Australia, Australia; 4 Stroke Unit, Royal Perth Hospital, Perth, Western Australia, Australia; 5 School of Medicine and Pharmacology, University of Western Australia, Perth, Western Australia, Australia; 6 Department of Geriatric Medicine, Royal Perth Hospital, Perth, Western Australia, Australia; University of Maryland School of Pharmacy, United States of America

## Abstract

**Context:**

Depression is associated with increased mortality, but it is unclear if this relationship is dose-dependent and if it can be modified by treatment with antidepressants.

**Objective:**

To determine if (1) the association between depression and mortality is independent of other common potential causes of death in later life, (2) there is a dose-response relationship between increasing severity of depression and mortality rates, and (3) the use of antidepressant drugs reduces mortality rates.

**Methods:**

Cohort study of 5,276 community-dwelling men aged 68–88 years living in Perth, Australia. We used the Geriatric Depression Scale 15-items (GDS-15) to ascertain the presence and severity of depression. GDS-15≥7 indicates the presence of clinically significant depression. Men were also grouped according to the severity of symptoms: “no symptoms” (GDS-15 = 0), “questionable” (1≤GDS-15≤4), “mild to moderate” (5≤GDS-15≤9), and “severe” (GDS-15≥10). Participants listed all medications used regularly. We used the Western Australian Data Linkage System to monitor mortality.

**Results:**

There were 883 deaths between the study assessment and the 30th June 2008 (mean follow-up of participants: 6.0±1.1 years). The adjusted mortality hazard (MH) of men with clinically significant depression was 1.98 (95%CI = 1.61–2.43), and increased with the severity of symptoms: 1.39 (95%CI = 1.13–1.71) for questionable, 2.71 (95%CI = 2.13–3.46) for mild/moderate, and 3.32 (95%CI: 2.31–4.78) for severe depression. The use of antidepressants increased MH (HR = 1.31, 95%CI = 1.02–1.68). Compared with men who were not depressed and were not taking antidepressants, MH increased from 1.22 (95%CI = 0.91–1.63) for men with no depression who were using antidepressants to 1.85 (95%CI = 1.47–2.32) for participants who were depressed but were not using antidepressants, and 2.97 (95%CI = 1.94–4.54) for those who were depressed and were using antidepressants. All analyses were adjusted for age, educational attainment, migrant status, physical activity, smoking and alcohol use and the Charlson comorbidity index.

**Conclusions:**

The mortality associated with depression increases with the severity of depressive symptoms and is largely independent of comorbid conditions. The use of antidepressants does not reduce the mortality rates of older men with persistent symptoms of depression.

## Introduction

Depression is a common and disabling condition that affects 1 in 6 people over the course of their lives [1], [2]. Its presence has been associated with deteriorating physical health [3] and increased mortality [4], [5], [6], [7], [8]. although the mechanisms linking depression to these poor health outcomes remain unclear. Smoking, obesity and physical inactivity are more prevalent amongst depressed than non-depressed adults, and may explain, at least in part, this excess in morbidity and mortality [9], [10], [11], [12]. There is a similarly higher prevalence of hypertension and diabetes amongst people with depression [13], [14], which together with deficient immune and inflammatory responses [15], may contribute to further increments in ill health and death.

Antidepressant treatment could conceivably minimise the negative health implications associated with depression by acting directly on the physiological pathways that leads to greater morbidity and mortality or, indirectly, by improving compliance with the management of comorbid disorders and facilitating a healthier lifestyle [16], [17]. A secondary analysis of the Enhancing Recovery in Coronary Heart Disease (ENRICHD) study showed that treatment with selective serotonin reuptake inhibitors (SSRIs) reduces subsequent cardiovascular morbidity and mortality in adults who were depressed after they had a heart attack [18]. Treatment with SSRIs has also been associated with a lower incidence rate ratio of colorectal cancer over a period of 5 years in a nested case-control study [19], although there are lingering concerns about the potential carcinogenic effects of some antidepressants [20], [21], [22]. In addition, there is evidence from ecological studies that the rise in the use of antidepressants has been associated with a concomitant decline in the number of suicides in the community [23], but it is debatable if the link between antidepressant sales and death by suicide is truly causal [24].

Results from the Three City Study (3C) [25] suggest that treatment with antidepressants may not necessarily reduce the risk of death in older adults with depression; in fact, the opposite may be true. The 3C was a longitudinal study that followed 7,363 French men and women aged 65 years or over for 4 years. Participants were screened with the Centre for Epidemiological Studies Depression Scale (CES-D) and interviewed with the Mini International Neuropsychiatric Interview (MINI) to establish the diagnosis of major depression according to DSM-IV criteria. Older adults with a CES-D score between 16 and 22 (inclusive) who did not meet criteria for major depression were considered to have ‘mild depression’, whereas participants with major depression or a CES-D score of 23 or more were considered to have ‘severe depression’. Men and women with severe depression not treated with antidepressants had an 80% higher mortality hazard than participants with no depression who were not using antidepressants. The mortality hazard increased for men, but not women, with mild (hazard ratio [HR] = 2.8, 95% confidence interval [CI] = 1.0–7.7) and severe depression treated with antidepressants (HR = 5.3, 95%CI = 2.7–10.5). However, the number of deaths in this investigation was small (215 men and 165 women) and the study lacked power to establish unequivocally the role of antidepressants on mortality.

We designed the present study to determine: (1) whether older men with clinically significant depressive symptoms have increased mortality, (2) whether the association between depression and mortality in older men is independent of other common potential causes of mortality in later life, (3) whether there is a dose-response relationship between increasing severity of depression and mortality rates, (4) whether the severity of depression influences the cause of death, and (5) whether the use of antidepressant drugs is associated with a decline in mortality rates.

## Methods

### Study design and participants

This study used a population-based sample of older men living in the Perth metropolitan area that represents the inception cohort of the Health In Men Study (HIMS). Details regarding the recruitment of participants have been described elsewhere [26]. During the years 2001–2004 those men who were still alive were contacted and invited to complete a follow-up assessment. We excluded from the present study men with a recorded history of dementia, drug abuse or dependence, psychosis or depression before 2001 – their exclusion aimed to minimize confounding associated with the past use of psychotropic agents, in particular antipsychotics [27]. These diagnoses were established by means of the Western Australian Data Linkage System (WADLS), which connects together all death records, acute hospital admissions, hospital movements, cancer registry, as well as psychiatric outpatient contacts for all residents of Western Australia since 1980 [28], [29]. This report refers to 5,276 subjects who were eligible and consented to follow-up. The Human Research Ethics Committee of the University of Western Australia approved the study protocol and all subjects provided written informed consent to participate.

### Outcome of interest

All cause mortality was the primary outcome of interest of the study. The Western Australian Data Linkage System (WADLS) retrieved information from the Australian Bureau of Statistics that reported the cause(s) of death of participants according to the ICD-10 (http://www.abs.gov.au/ausstats/abs.nsf/mf/3303.0).

### Explanatory variables

Participants were asked to complete the 15-item Geriatric Depression Scale (GDS-15) at the baseline assessment and, *a priori,* those with a total score of 7 or more were considered to display clinically significant depressive symptoms at the time of assessment. This relatively high cut-point was chosen to ensure high specificity for the diagnosis of depression in this sample [30]. We also used previously published data to guide the grouping of participants according to the severity of depressive symptoms: ‘no’ (GDS-15 total score = 0), ‘questionable’ (GDS-15 total score 1 to 4), ‘mild to moderate’ (GDS-15 total score 5 to 9) and ‘severe’ depression (GDS-15 total score 10 to 15) [30].

We calculated the age of participants as the difference in days between the date of the assessment and their date of birth divided by 365.25. In addition, men reported the highest level of education achieved (completed or not high school) and their country of birth. Participants were also asked whether they were currently smoking cigarettes, cigars or a pipe regularly (possible answers: every day/not every day/not at all). Men who answered ‘every day’ or ‘not every day’ were classified as current smokers.

At the time of assessment, we asked men if they had been told by a doctor during the previous 5 years that they had arthritis, cancer (colon, prostate or melanoma), a cardiovascular illness (angina, myocardial infarction or stroke), diabetes, hypertension, or pulmonary diseases (chronic bronchitis, emphysema or asthma). We then used administrative medical information from WADLS during the 10 years prior to assessment at HIMS to calculate the Charlson index and determine the presence of significant medical comorbidity in our sample [31]. The index takes into account 17 common medical conditions that predict 1-year mortality: myocardial infarction, congestive heart failure, peripheral arterial disease, cerebrovascular disease, dementia, chronic pulmonary disease, connective tissue disease, ulcer disease, liver disease, diabetes (including diabetes with end organ damage), hemiplegia, renal disease, leukemia, lymphoma, other tumours, metastatic tumours and AIDS. Charlson and colleagues used adjusted relative risks to assign integer weights to these conditions within a composite index score that ranges from 0 to 37. Coding algorithms to define comorbidities followed the procedures described by Quan et al. [32] and scores were calculated using Stagg's Charlson's index Stata 9.2 routine (StataCorp, College Station, Texas). We stratified scores, as per usual practice [33], to reflect the increasing severity of comorbidity associated with the index.

Finally, we asked participants to list all medications they were consuming on a regular basis at the time of assessment at HIMS. We coded the medications according to the World Health Organisation Standard Classification of Medications [34]. Only information about the use of antidepressants was retrieved for this study.

### Statistical analyses

Data were analyzed with the statistical package Stata release 10.0 (StataCorp, College Station, TX). We used 2×2 tables to calculate the crude risk ratio of death amongst study participants and non-participants. We identified potential confounders of the association between depression and mortality by comparing men with and without depression for the distribution of relevant demographic, lifestyle and clinical variables. After completing the preliminary univariate analysis, Cox proportional-hazards regression models were applied to estimate the adjusted mortality hazard associated with prevalent depression. We progressively included demographic, lifestyle and clinical variables, and retained in the final model only those that contributed significantly to mortality (age was entered in the adjusted models as a continuous variable). Depression was included in the models as a binary (GDS≥7, yes/no) or ordinal variable (low, questionable, mild/moderate and severe depressive symptoms).

We grouped participants into four categories to investigate the interaction between antidepressant use, depression and mortality: no depression and no antidepressants, no depression with antidepressants, depression and no antidepressants, and depression with antidepressants. Taking the first group as reference, we used Cox regression models to assess the association between group membership and mortality, and adjusted all analyses for the relevant demographic, lifestyle and clinical risk factors.

Finally, we used a 2×2 tables to calculate the absolute risk increase as well as the number needed to harm associated with the use of antidepressant treatment of older men with depression. Ninety-five percent confidence intervals (95%CI) were estimated for the risk ratio, hazard ratio, absolute risk increase, and number needed to harm. Alpha was set at 5% and all probability tests reported are two-tailed.

We estimated that a study with 5,276 participants would have 80% power to declare as significant an adjusted mortality hazard ratio of 1.25 or greater, as estimated by the Stata command stpower cox.

## Results

**Figure 1:**
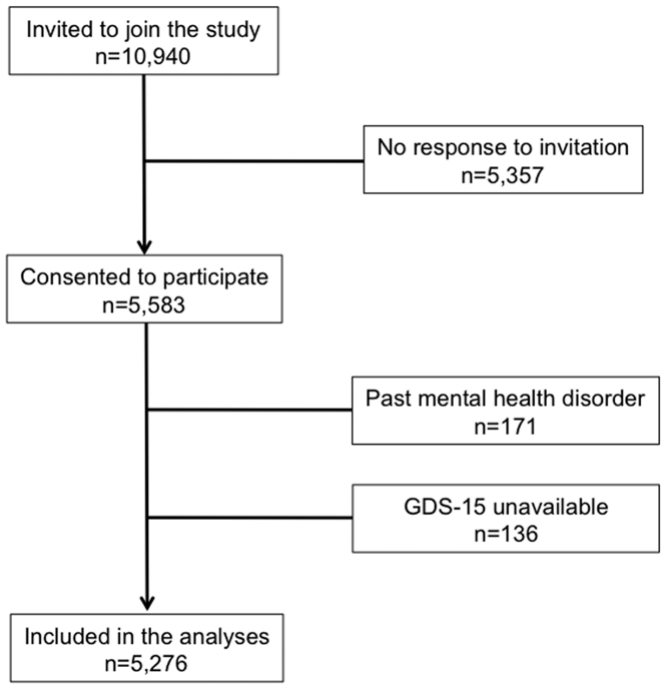
The diagram shows the flow of participants from the time of invitation to inclusion in the study.

Figure 1 shows the flow of participants from invitation to inclusion in this study. The age of participating men ranged from 68 to 88 years (mean±SD: 76.7±3.8) and 297 (5.6%) of them displayed clinically significant symptoms of depression at the time of assessment (i.e., GDS-15≥7). Participants with depression were older than non-depressed men (t = 2.74, df = 5274, p = 0.006), although the age difference between the groups was small (77.3±3.8 vs 76.7±4.0, respectively). Older men with depression had greater odds of having been born overseas or having ever smoked, drinking 28 or more standard drinks of alcohol over a usual one-week period, having a body mass index greater than 25 (i.e., overweight or obese) and showing evidence of greater medical morbidity. In contrast, physical activity (vigorous and non-vigorous) and higher education were associated with reduced odds of depression. Table 1 summarises the demographic, lifestyle and medical characteristics of participants with and without depression.

**Table 1:** Demographic, lifestyle and clinical factors associated with prevalent depression.

	DepressedN = 297n (%)	Not depressedN = 4,979n (%)	Odds Ratio (95%CI)
Demographics			
Completed at least high school	106 (35.7)	2,320 (46.6)	0.63 (0.50–0.81)
Born overseas	139 (46.8)	1,951 (39.2)	1.37 (1.08–1.73)
Lifestyle			
Current smoking	26 (8.7)	242 (4.9)	1.88 (1.18–2.89)
Comorbidities			
Arthritis	141 (47.5)	1,629 (32.7)	1.86 (1.49–2.36)
Cancer*	63 (21.2)	692 (13.9)	1.67 (1.25–2.29)
Cardiovascular disease#	113 (38.1)	975 (19.6)	2.52 (1.97–3.23)
Diabetes	59 (19.9)	525 (10.5)	2.10 (1.56–2.84)
Hypertension	101 (34.0)	1,482 (29.8)	1.22 (0.95–1.56)
Pulmonary diseases$	69 (23.2)	558 (11.2)	2.40 (1.80–3.19)
Charlson index (weighted) 01–23–45+	162 (54.6)48 (16.2)53 (17.9)34 (11.5)	3,686 (74.0)645 (13.0)436 (8.8)212 (4.3)	11.69 (1.21–2.36)2.77 (2.00–3.83)3.65 (2.46–5.42)
Antidepressants			
Any	52 (17.5)	247 (5.0)	4.10 (2.88–5.67)
Tricyclic antidepressants	17 (5.7)	110 (2.2)	2.69 (1.49–4.58)
SSRIs	26 (8.8)	109 (2.2)	4.29 (2.63–6.75)
Other antidepressants	12 (4.0)	31 (0.6)	6.72 (3.11–13.62)

SSRIs = selective serotonin reuptake inhibitors.

*melanoma, colon and prostate cancer;

# angina, myocardial infarction and stroke;

$ chronic bronchitis, emphysema and asthma.

There were 883 deaths between the study assessment and the 30^th^ June 2008 (mean follow-up of participants: 6.0±1.1 years). The characteristics of men who died during the follow up period are described in table 2. The relative risk of death amongst men with clinically significant depression was 2.26 (95%CI: 1.91–2.53) compared with non-depressed men. The risk of death was higher in the older age group, as well as amongst men who had not completed high school education, who had ever smoked, drank more than 28 standard drinks of alcohol per week, did not engage in regular vigorous physical activity, or who had cancer, cardiovascular disease, diabetes and diseases of the lungs. The risk of death increased with the severity of clinical comorbidity, as measured by the Charlson index.

**Table 2:** Demographic, lifestyle and clinical factors associated with death during follow up.

	DeadN = 883n (%)	AliveN = 4,393n (%)	Relative Risk (95%CI)
Depression	105 (11.9)	192 (4.4)	2.26 (1.91–2.53)
Demographics			
Age at entry, mean (sd)	78.7 (4.2)	76.3 (3.6)	2.37 (2.10–2.64)
Completed at least high school	357 (40.4)	2,069 (47.1)	0.81 (0.72–0.91)
Born overseas	341 (38.6)	1,749 (39.8)	0.95 (0.84–1.07)
Lifestyle			
Current smoking	73 (8.3)	195 (4.4)	1.94 (1.45–2.58)
Comorbidities			
Arthritis	322 (35.5)	1,475 (33.5)	1.08 (0.95–1.22)
Cancer*	176 (19.2)	588 (13.3)	1.42 (1.23–1.64)
Cardiovascular disease#	269 (29.3)	8.47 (19.2)	1.57 (1.38–1.78)
Diabetes	119 (13.1)	475 (10.8)	1.19 (1.00–1.42)
Hypertension	272 (29.8)	1,332 (30.6)	0.98 (0.86–1.12)
Pulmonary diseases$	115 (19.7)	427 (13.2)	1.66 (1.43–1.92)
Charlson index (weighted) 01–23–45+	493 (55.8)148 (16.6)144 (16.3)98 (11.1)	3,355 (76.4)545 (12.4)345 (7.9)148 (3.4)	11.84 (1.51–2.27)2.84 (2.29–3.52)4.51 (3.43–5.92)
Antidepressants			
AnyTCASSRIsOther	70 (7.9)30 (3.4)32 (3.6)9 (1.0)	229 (5.2)97 (2.2)103 (2.3)34 (0.8)	1.57 (1.16–2.08)1.56 (0.99–2.38)1.57 (1.01–2.37)1.32 (0.55–2.82)

SSRIs = selective serotonin reuptake inhibitors.

*melanoma, colon and prostate cancer;

# angina, myocardial infarction and stroke;

$ chronic bronchitis, emphysema and asthma.

We used Cox regression to determine if the increased risk of death associated with depression could be explained by other measured factors. The crude rate ratio of death associated with depression was 2.58 (95%CI: 2.11–3.17), and was reduced to 2.40 (95%CI: 1.95–2.94) after the analyses were adjusted for age, educational attainment, and migrant status. Additional adjustments including current smoking (rate ratio: 2.32, 95%CI: 1.89–2.85), and self-reported comorbidities (rate ratio: 2.07, 95%CI: 1.67–2.55) or the Charlson index (rate ratio: 1.98, 95%CI: 1.61–2.43) showed that the excess mortality associated with depression could not be adequately explained by these factors.

**Figure 2:**
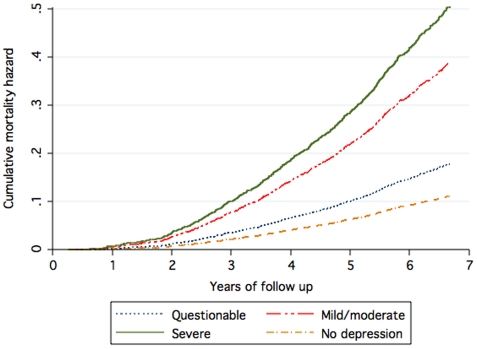
Cumulative mortality hazard over time (in years) associated with the severity of depressive symptoms (adjusted for all measured demographic and lifestyle factors as well as comorbidities listed in **table 1**). The groups with ‘no’, ‘questionable’, ‘mild to moderate’ and ‘severe’ depression corresponded to GDS-15 total scores of 0 (n = 1,166), 1 to 4 (n = 3,465), 5 to 9 (n = 533) and 10 or greater (n = 112), respectively.

We then conducted a series of analyses to clarify if death in this cohort was associated with the severity of depressive symptoms. One thousand one hundred and sixty-six men reported no depressive symptoms at assessment (total GDS-15 score = 0) and were used as the reference group in a fully adjusted Cox regression model that included all measured demographic, lifestyle and comorbid variables (measured by the Charlson index). Figure 2 illustrates the results of these analyses. The rate ratio of death associated with depression increased with the severity of symptoms: 1.39 (95%CI: 1.13–1.71) for questionable, 2.71 (95%CI: 2.13–3.46) for mild/moderate, and 3.32 (95%CI: 2.31–4.78) for severe depression, respectively.

Table 3 summarises the most frequent causes of death according to the severity of depressive symptoms. The commonest cause of death amongst participants with no depressive symptoms at the baseline assessment was cancer, whereas cardiovascular diseases were the most prevalent contributors to the death of men with mild to severe depressive symptoms. Posthoc analyses showed that, compared with non-depressed participants, the hazard of death due to cardiovascular disease increased to 1.52 (95%CI = 1.10–2.11) for questionable depression, and 4.36 (95%CI = 3.03–6.27) and 5.71 (95%CI = 3.43–9.50) for mild/moderate and severe depression, respectively. (All analyses were adjusted for age at entry, education level, country of birth and smoking status).

**Table 3:** Mortality rates (95% confidence interval) per 1,000 persons per year according to the severity of depressive symptoms at the time of assessment.

Causes of death, n (%)	No depression*N = 110 deaths	Questionable depression#N = 556 deaths	Mild/moderate depression+N = 176 deaths	Severe depression‡N = 41 deaths
Cardiovascular diseases	6.1 (4.4–8.5)	9.2 (7.7–11.1)	25.5 (20.0–32.6)	32.4 (21.1–50.0)
Cancer	7.7 (5.7–10.3)	9.9 (8.2–11.9)	15.1 (11.2–20.4)	15.2 (8.3–28.0)
Chronic respiratory diseases	0.9 (0.4–2.0)	2.0 (1.4–3.0)	5.8 (3.5–9.6)	7.8 (3.3–18.4)
Infections	3.3 (2.1–5.1)	5.5 (4.3–7.0)	10.6 (7.4–15.3)	12.8 (6.5–25.2)
Dementia	0.3 (0.1–1.1)	1.3 (0.8–2.2)	5.5 (3.2–9.4)	6.5 (2.5–16.8)
Suicide or accidents	0.0 (0.0–0.7)	0.0 (0.0–0.3)	0	0
Other causes	0.8 (0.3–2.0)	1.2 (0.7–2.1)	1.2 (0.4–3.4)	0

Contributing causes of death for each depression group were

*135,

# 723,

+ 243 and

‡ 54 causes of death (i.e., death might have been attributed to more than one cause).

All rates were standardised for age (median age of 76.3 years), education, country of birth and smoking status.

**Figure 3:**
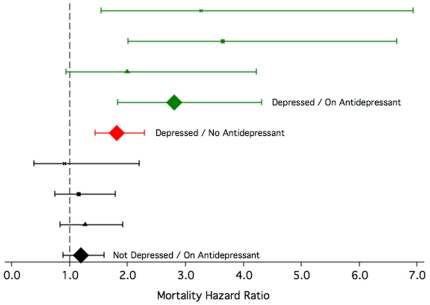
Mortality hazard of men without depression treated with antidepressants (n = 247, black), men with depression not treated with antidepressants (n = 245, red), and men with depression treated with antidepressants (n = 52, green) (reference group: men with no depression not using antidepressants, n = 4,732). The mortality hazard ratio associated with the use tricyclic antidepressants (▴), selective serotonin inhibitors (SSRIs)(▪), other antidepressants (x) and all antidepressants combined (⧫) is shown in the figure. The whiskers represent the 95% confidence interval of the mortality hazard ratio. All analyses were adjusted for the demographic and lifestyle factors, as well as the comorbidities listed in table 1.

Figure 3 shows the mortality hazard of men according to whether or not they were using antidepressants at the beginning of the follow up period. We used as the reference group men who showed no evidence of clinically significant depression (i.e., GDS-15<7) and who were not taking antidepressants. The mortality hazard ratio increased to 1.22 (95%CI = 0.91–1.63) for men with no depression who were using antidepressants, 1.85 (95%CI = 1.47–2.32) for participants who were depressed but were not using antidepressants, and 2.97 (95%CI = 1.94–4.54) for those who were depressed and were using antidepressants.

The mortality hazard associated with the interaction between depression and antidepressant use was 1.32 (95%CI = 0.76–2.30; z = 0.98, p = 0.328). As the interaction between depression and antidepressant use did not contribute to explain mortality, the term was not included in the final explanatory model, which showed that the independent (fully adjusted) mortality hazard associated with depression and antidepressant use was 1.93 (95%CI = 1.57–2.38; z = 6.18, p<0.001) and 1.31 (95%CI = 1.02–1.68; z = 2.14, p = 0.032), respectively.

Table 4 summarises the most frequent causes of death amongst these men, whereas table 5 lists the most frequent causes of death according to the type of antidepressant used by participants. We conducted a series of analyses to determine if depression and antidepressants were associated with cause-specific mortality. Both depression (HR = 2.69, 95%CI = 2.01–3.59; z = 6.67, p<0.001) and antidepressant use (HR = 1.43, 95%CI = 1.00–2.03; z = 1.99, p = 0.047) were independently associated with death due to cardiovascular disease after adjustments were made for age, education, country of birth and smoking. Depression was also associated with greater adjusted mortality hazard due to cancer (HR = 1.76, 95%CI = 1.23–2.52; z = 3.10, p = 0.002), but not antidepressants (HR = 1.01, 95%CI = 0.65–1.56; z = 0.05, p = 0.960). No further analyses were conducted because of the relatively small number of deaths due to other causes.

**Table 4:** Mortality rates (95% confidence interval) per 1,000 persons per year according to depression (GDS-15≥7) and antidepressant use.

Causes of death, n (%)	No depression and no antidepressant*N = 730 deaths	No depression with antidepressant#N = 48 deaths	Depression and no antidepressant+N = 83 deaths	Depression with antidepressant‡N = 22 deaths
Cardiovascular diseases	9.5 (8.0–11.3)	11.9 (7.6–18.6)	22.9 (16.3–32.2)	41.8 (24.4–71.7)
Cancer	9.8 (8.2–11.6)	7.5 (4.3–13.2)	14.2 (9.3–21.73)	27.0 (13.9–52.6)
Chronic respiratory diseases	1.9 (1.3–2.8)	2.2 (0.8–6.0)	8.2 (4.5–15.0)	8.6 (2.7–27.4)
Infections	5.2 (4.1–6.5)	6.0 (3.2–11.1)	11.6 (7.2–18.7)	20.5 (9.6–43.8)
Dementia	1.4 (0.9–2.2)	1.0 (0.2–4.1)	5.1 (2.4–10.5)	8.0 (2.4–26.1)
Suicide or accidents	0.0 (0.0–0.3)	0.1 (0.0–2.6)	0	0
Other causes	1.1 (0.6–1.8)	2.5 (0.9–7.3)	0	0

Contributing causes of death for each group were

*860,

# 55,

+ 104 and

‡ 34 causes of death (i.e., death might have been attributed to more than one cause).

All rates were standardised for age (median age of 76.3 years), education, country of birth and smoking status.

Finally, we completed sensitivity analyses to determine if the association between depression, antidepressants and mortality had been confounded by severity of physical illness around the time of assessment at HIMS, such that poor physical health could have led to depressive symptoms at HIMS and death at follow up. We repeated the analyses described above after excluding the 22 men who died during the first year of follow up. Clinically significant depression (GDS-15≥7) continued to be associated with increased mortality hazard (HR = 1.95, 95%CI = 1.58–2.41; z = 6.22, p<0.001), as did the use of antidepressants (HR = 1.30, 95%CI = 1.01–1.66; z = 2.04, p = 0.041). We extended our sensitivity analyses by excluding from the analyses all men who died during the first 2 years of follow up (n = 75). Again, we observed a consistent increase in the mortality hazard associated with depression (HR = 1.92, 95%CI = 1.54–2.39; z = 5.78, p<0.001) and antidepressant use (HR = 1.30, 95%CI = 1.00–1.69; z = 1.98, p = 0.047). All analyses were adjusted for the factors listed in table 1.

**Table 5:** Mortality rates (95% confidence interval) per 1,000 persons per year according to antidepressant group use.

Causes of death, n (%)	No antidepressant*N = 813 deaths	TCAs#N = 27 deaths	SSRIs^+^N = 32 deaths	Other antidepressants‡N = 11 deaths
Cardiovascular diseases	10.1 (8.5–12.0)	15.4 (9.0–26.6)	17.1 (10.5–28.1)	19.3 (8.0–46.7)
Cancer	10.0 (8.4–11.9)	11.3 (6.0–21.3)	10.5 (5.6–19.8)	3.8 (0.5–27.4)
Chronic respiratory diseases	2.2 (1.5–3.1)	4.1 (1.5–11.4)	1.9 (0.5–7.7)	7.7 (1.9–31.2)
Infections	5.4 (4.3–6.8)	3.2 (1.0–10.0)	11.6 (6.4–21.1)	8.0 (2.0–32.1)
Dementia	1.6 (1.0–2.4)	0	3.5 (1.2–9.8)	3.4 (0.5–25.2)
Suicide or accidents	0.1 (0.0–2.1)	0.3 (0.0–5.7)	0	0
Other causes	1.0 (0.6–1.8)	2.5 (0.6–10.7)	1.1 (0.2–8.6)	4.0 (0.5–30.0)

TCAs = tricyclic antidepressants, SSRIs =  selective serotonin reuptake inhibitors. Contributing causes of death for each group were.

*1063,

# 34,

+ 46 and

‡ 16 causes of death (i.e., death might have been attributed to more than one cause).

All rates were standardised for age (median age of 76.3 years), education, country of birth and smoking status.

## Discussion

The results of this study indicate that the 6-year adjusted mortality hazard is twice as high for men with depression compared with non-depressed men and that the use of antidepressants is associated with an independent rise in mortality of 30%. Our findings also show that the rate ratio of death increases with the severity of depressive symptoms, and that the causes of death of men with mild to severe depression are different from men with no or questionable depression: cardiovascular diseases are more prevalent amongst depressed men, whereas cancer is slightly more frequent amongst men with no or questionable depression. Finally, we found that the adjusted mortality hazard of participants increased progressively according to depression status and antidepressant use, being nearly 3 times as high for men with depression on antidepressants compared with non-depressed men not using antidepressants, and 1.8 for men with depression not using antidepressants.

Before discussing the implications of our findings, we wish to highlight some of the strengths and limitations of the study design and place them into context. This project has the merit of having used a well established cohort of older men for whom detailed clinical and administrative information is available [26]. The assessment at HIMS allowed us to collect information about the severity of depressive symptoms and the use of antidepressants at the time of entry into the study, whilst WADLS provided systematic information about mortality and its causes. As moves into and away from Western Australia are negligible in this age group [35], WADLS was able to track the deaths that occurred in the entire cohort during the follow up period with great accuracy. In addition, WADLS is one of the most robust systems to monitor the occurrence of health events in the population currently available in the World [29], and this enabled us to ascertain the independent contribution of depression to the mortality of older men without having to rely on self-reported information about the presence of various comorbid conditions. In addition, as WADLS provides complete information on mortality for the entire population, there was no differential loss to follow up of people with and without depression. Nonetheless, our ascertainment of the exposures of interest (i.e., depression and antidepressant treatment) was limited to the starting point of the study, and we are therefore unable to comment on the likely impact that changes in the level of these exposures during the follow up period could have had on mortality.

We also acknowledge that the assessment of depression was not based on a structured clinical interview and, as a result, our data do not allow for direct inferences to be made about the differential 6-year mortality of older men with a DSM-IV episode of major depression, although the approach that we used has been previously shown to have good sensitivity and specificity for the diagnosis of major depression [30]. Another limitation of our survey is that it was limited to men and our results may not necessarily apply to women. Furthermore, we acknowledge that the observational nature of the study and the inherent non-randomised comparison of older men with depression who were or not treated with antidepressants is subject to bias, especially confounding by indication, whereby unmeasured factors (which we were consequently unable to adjust for in our analyses) might have influenced the choice of antidepressant treatment and contributed to subsequent mortality. Finally, we attempted to minimise the effect that significant medical morbidity at the time of assessment could have had on the expression of depressive symptoms [36] and mortality by repeating our analyses after the exclusion of participants who had died during the first and second years of follow up, and found that the results of the study were not altered. This, together with the observation that older men with depression seem less likely to die from cancer (a group of diseases that is associated with high lethality), suggests that the observed associations in our study cannot be easily dismissed as a result of confounding and bias.

Given the characteristics of the study, we would argue that our findings of increased mortality associated with depression in older men are valid: they are twice as likely to die over a 6-year period as non-depressed men. The results of previous studies investigating the mortality of older people with depression lend further support to our conclusions and are summarised in Table S1 (citations 50 to 62 [50]–[62] refer to studies listed in this table. Please see also review by Cuijpers et al. [37]). In addition, we found a dose-response relationship between the severity of depressive symptoms and death in our cohort, with the mortality hazard of men with severe depression being more than three-times higher than that of non-depressed participants. Two studies had previously attempted to investigate the association between the severity of depression and death. The most recent was based on the Amsterdam Study of the Elderly (AMSTEL) and included 1,989 people aged 65–84 years that were followed for up to 10 years [38]. They found that moderate and severe depression were associated with 29% (95%CI = 3%–61%) and 34% (95%CI = 7%–68%) increase in mortality hazard compared with non-depressed older people, but were unable to demonstrate a clear dose effect of depression partly because of the large number of participants lost during follow up (about 4,000 people). Loss to follow up is a common problem of longitudinal studies that we were able to minimise by using the WADLS. The 3C study [25], which we have previously described, found a dosage relationship between the severity of depressive symptoms and death amongst men taking antidepressants, but not amongst those with depression not using antidepressants, a finding that is consistent with our own results.

We found that antidepressant treatment increases the mortality hazard of men by 30%, and this association is independent of the presence of clinically significant depression. However, it is possible that non-surviving men had a more chronic and unremitting course of illness, which may have confounded the direct effect of antidepressants on mortality. Such an interpretation of our findings is partly supported by the lack of association between antidepressant use and mortality amongst men without depression, and suggests that the higher mortality associated with depression decreases if treatment is associated with the remission of symptoms. Heart rate variability (HRV) is one of the possible physiological mechanisms linking depression, antidepressants and death. There is increasing evidence that adults with depression have reduced HRV (i.e., decrease ability to increase/decrease heart rate according to physiological demands) and that reduced HRV increases mortality [39], [40]. Recovery from depression (spontaneously or as a result of treatment) increases HRV [41] and this may contribute to reduce mortality amongst adults at risk. A recent review of this topic confirms that this is a plausible explanation for our results [42].

It is also important to consider that the use of antidepressants has been associated with numerous potentially harmful effects, some of which may increase morbidity and mortality. For example, antidepressant treatment has been linked to increased risk of injurious and non-injurious falls in cross-sectional and longitudinal studies [43], [44], and there is some evidence that the use of common antidepressants increases the risk bleeding in various body systems, including the central nervous system [45], as well as the risk of incident diabetes [46]. In addition, recently published findings from the Nurses' Health Study showed an increase in the number of sudden cardiac deaths associated with the use of antidepressants [47], a result that is consistent with our observation of an excess of cardiovascular deaths amongst men using antidepressants. Although these results cannot be considered conclusive at this stage, as mortality information from long-term randomized trials of antidepressants is not yet available, they do highlight the need for further research and caution when prescribing antidepressants to vulnerable older adults, particularly when one considers that the efficacy of these drugs is modest [48], [49].

In summary, the results of our study indicate that mortality hazard increases with the severity of depressive symptoms and that treatment with antidepressants fails to reduce the mortality rate of older men with persistent symptoms of depression.

## Supporting Information

Table S1 (0.16 MB DOC)

## References

[1] Prince M, Patel V, Saxena S, Maj M, Maselko J (2007). No health without mental health.. Lancet.

[2] Kessler RC, Berglund P, Demler O, Jin R, Koretz D (2003). The epidemiology of major depressive disorder: results from the National Comorbidity Survey Replication (NCS-R).. JAMA.

[3] Tiemeier H, Breteler MM, Hofman A, Stijnen T (2005). A multivariate score objectively assessed health of depressed elderly.. J Clin Epidemiol.

[4] Surtees PG, Wainwright NW, Luben RN, Wareham NJ, Bingham SA (2008). Depression and ischemic heart disease mortality: evidence from the EPIC-Norfolk United Kingdom prospective cohort study.. Am J Psychiatry.

[5] Ensinck KT, Schuurman AG, van den Akker M, Metsemakers JF, Kester AD (2002). Is there an increased risk of dying after depression?. Am J Epidemiol.

[6] Penninx BW, Geerlings SW, Deeg DJ, van Eijk JT, van Tilburg W (1999). Minor and major depression and the risk of death in older persons.. Arch Gen Psychiatry.

[7] Zheng D, Macera CA, Croft JB, Giles WH, Davis D (1997). Major depression and all-cause mortality among white adults in the United States.. Ann Epidemiol.

[8] Schoevers RA, Geerlings MI, Beekman AT, Penninx BW, Deeg DJ (2000). Association of depression and gender with mortality in old age. Results from the Amsterdam Study of the Elderly (AMSTEL).. Br J Psychiatry.

[9] Almeida OP, Pfaff JJ (2005). Depression and smoking amongst older general practice patients.. J Affect Disord.

[10] Cassidy K, Kotynia-English R, Acres J, Flicker L, Lautenschlager NT (2004). Association between lifestyle factors and mental health measures among community-dwelling older women.. Aust N Z J Psychiatry.

[11] Blumenthal JA, Babyak MA, Moore KA, Craighead WE, Herman S (1999). Effects of exercise training on older patients with major depression.. Arch Intern Med.

[12] Simon GE, Von Korff M, Saunders K, Miglioretti DL, Crane PK (2006). Association between obesity and psychiatric disorders in the US adult population.. Arch Gen Psychiatry.

[13] Almeida OP, Flicker L, Norman P, Hankey GJ, Vasikaran S (2007). Association of cardiovascular risk factors and disease with depression in later life.. Am J Geriatr Psychiatry.

[14] Maraldi C, Volpato S, Penninx BW, Yaffe K, Simonsick EM (2007). Diabetes mellitus, glycemic control, and incident depressive symptoms among 70- to 79-year-old persons: the health, aging, and body composition study.. Arch Intern Med.

[15] Dantzer R, O'Connor JC, Freund GG, Johnson RW, Kelley KW (2008). From inflammation to sickness and depression: when the immune system subjugates the brain.. Nat Rev Neurosci.

[16] Cox LS, Patten CA, Niaura RS, Decker PA, Rigotti N (2004). Efficacy of bupropion for relapse prevention in smokers with and without a past history of major depression.. J Gen Intern Med.

[17] Katon W, Cantrell CR, Sokol MC, Chiao E, Gdovin JM (2005). Impact of antidepressant drug adherence on comorbid medication use and resource utilization.. Arch Intern Med.

[18] Taylor CB, Youngblood ME, Catellier D, Veith RC, Carney RM (2005). Effects of antidepressant medication on morbidity and mortality in depressed patients after myocardial infarction.. Arch Gen Psychiatry.

[19] Xu W, Tamim H, Shapiro S, Stang MR, Collet JP (2006). Use of antidepressants and risk of colorectal cancer: a nested case-control study.. Lancet Oncol.

[20] Dalton SO, Johansen C, Mellemkjaer L, Sorensen HT, McLaughlin JK (2000). Antidepressant medications and risk for cancer.. Epidemiology.

[21] Brandes LJ, Arron RJ, Bogdanovic RP, Tong J, Zaborniak CL (1992). Stimulation of malignant growth in rodents by antidepressant drugs at clinically relevant doses.. Cancer Res.

[22] Dalton SO, Poulsen AH, Norgaard M, McLaughlin JK, Johansen C (2008). Tricyclic antidepressants and non-Hodgkin lymphoma.. Epidemiology.

[23] Gibbons RD, Hur K, Bhaumik DK, Mann JJ (2005). The relationship between antidepressant medication use and rate of suicide.. Arch Gen Psychiatry.

[24] Baldessarini RJ, Tondo L, Strombom IM, Dominguez S, Fawcett J (2007). Ecological studies of antidepressant treatment and suicidal risks.. Harv Rev Psychiatry.

[25] Ryan J, Carriere I, Ritchie K, Stewart R, Toulemonde G (2008). Late-life depression and mortality: influence of gender and antidepressant use.. Br J Psychiatry.

[26] Norman PE, Flicker L, Almeida OP, Hankey GJ, Hyde Z (2009). Cohort Profile: The Health In Men Study (HIMS).. Int J Epidemiol.

[27] Chahine LM, Acar D, Chemali Z (2010). The elderly safety imperative and antipsychotic usage.. Harv Rev Psychiatry.

[28] Holman CD, Bass AJ, Rouse IL, Hobbs MS (1999). Population-based linkage of health records in Western Australia: development of a health services research linked database.. Aust N Z J Public Health.

[29] Holman CD, Bass AJ, Rosman DL, Smith MB, Semmens JB (2008). A decade of data linkage in Western Australia: strategic design, applications and benefits of the WA data linkage system.. Aust Health Rev.

[30] Almeida OP, Almeida SA (1999). Short versions of the geriatric depression scale: a study of their validity for the diagnosis of a major depressive episode according to ICD-10 and DSM-IV.. Int J Geriatr Psychiatry.

[31] Charlson ME, Pompei P, Ales KL, MacKenzie CR (1987). A new method of classifying prognostic comorbidity in longitudinal studies: development and validation.. J Chronic Dis.

[32] Quan H, Sundararajan V, Halfon P, Fong A, Burnand B (2005). Coding algorithms for defining comorbidities in ICD-9-CM and ICD-10 administrative data.. Med Care.

[33] Almeida OP, McCaul K, Hankey GJ, Norman P, Jamrozik K (2008). Homocysteine and depression in later life.. Arch Gen Psychiatry.

[34] WHO (2005). World Health Organisation Collaborating Centre for Drug Statistics Methodology..

[35] ABS (2007).

[36] Pfaff JJ, Draper BM, Pirkis JE, Stocks NP, Snowdon JA (2009). Medical morbidity and severity of depression in a large primary care sample of older Australians: the DEPS-GP project.. Med J Aust.

[37] Cuijpers P, Smit F (2002). Excess mortality in depression: a meta-analysis of community studies.. J Affect Disord.

[38] Schoevers RA, Geerlings MI, Deeg DJ, Holwerda TJ, Jonker C (2009). Depression and excess mortality: evidence for a dose response relation in community living elderly.. International Journal of Geriatric Psychiatry.

[39] Bigger JT, Fleiss JL, Steinman RC, Rolnitzky LM, Kleiger RE (1992). Frequency domain measures of heart period variability and mortality after myocardial infarction.. Circulation.

[40] Carney RM, Blumenthal JA, Freedland KE, Stein PK, Howells WB (2005). Low heart rate variability and the effect of depression on post-myocardial infarction mortality.. Arch Intern Med.

[41] Glassman AH, Bigger JT, Gaffney M, Van Zyl LT (2007). Heart rate variability in acute coronary syndrome patients with major depression: influence of sertraline and mood improvement.. Arch Gen Psychiatry.

[42] Carney RM, Freedland KE (2009). Treatment-resistant depression and mortality after acute coronary syndrome.. Am J Psychiatry.

[43] Kerse N, Flicker L, Pfaff JJ, Draper B, Lautenschlager NT (2008). Falls, depression and antidepressants in later life: a large primary care appraisal.. PLoS ONE.

[44] Thapa PB, Gideon P, Cost TW, Milam AB, Ray WA (1998). Antidepressants and the risk of falls among nursing home residents.. N Engl J Med.

[45] Meijer WE, Heerdink ER, Nolen WA, Herings RM, Leufkens HG (2004). Association of risk of abnormal bleeding with degree of serotonin reuptake inhibition by antidepressants.. Arch Intern Med.

[46] Andersohn F, Schade R, Suissa S, Garbe E (2009). Long-term use of antidepressants for depressive disorders and the risk of diabetes mellitus.. Am J Psychiatry.

[47] Whang W, Kubzansky LD, Kawachi I, Rexrode KM, Kroenke CH (2009). Depression and risk of sudden cardiac death and coronary heart disease in women: results from the Nurses' Health Study.. J Am Coll Cardiol.

[48] Kirsch I, Deacon BJ, Huedo-Medina TB, Scoboria A, Moore TJ (2008). Initial severity and antidepressant benefits: a meta-analysis of data submitted to the Food and Drug Administration.. PLoS Med.

[49] Turner EH, Matthews AM, Linardatos E, Tell RA, Rosenthal R (2008). Selective publication of antidepressant trials and its influence on apparent efficacy.. N Engl J Med.

[50] Surtees PG, Wainwright NW, Luben RN, Wareham NJ, Bingham SA (2008). Psychological distress, major depressive disorder, and risk of stroke.. Neurology.

[51] Ahto M, Isoaho R, Puolijoki H, Vahlberg T, Kivela SL (2007). Stronger symptoms of depression predict high coronary heart disease mortality in older men and women.. Int J Geriatr Psychiatry.

[52] Gallo JJ, Bogner HR, Morales KH, Post EP, Lin JY (2007). The effect of a primary care practice-based depression intervention on mortality in older adults: a randomized trial.. Ann Intern Med.

[53] Ben-Ezra M, Shmotkin D (2006). Predictors of mortality in the old-old in Israel: the Cross-sectional and Longitudinal Aging Study.. J Am Geriatr Soc.

[54] Adamson JA, Price GM, Breeze E, Bulpitt CJ, Fletcher AE (2005). Are older people dying of depression? Findings from the Medical Research Council trial of the assessment and management of older people in the community.. J Am Geriatr Soc.

[55] van den Brink CL, Tijhuis M, van den Bos GA, Giampaoli S, Nissinen A (2005). The contribution of self-rated health and depressive symptoms to disability severity as a predictor of 10-year mortality in European elderly men.. Am J Public Health.

[56] Wulsin LR, Evans JC, Vasan RS, Murabito JM, Kelly-Hayes M (2005). Depressive symptoms, coronary heart disease, and overall mortality in the Framingham Heart Study.. Psychosom Med.

[57] Blazer DG, Hybels CF (2004). What symptoms of depression predict mortality in community-dwelling elders?. J Am Geriatr Soc.

[58] Wilson RS, Bienias JL, Mendes de Leon CF, Evans DA, Bennett DA (2003). Negative affect and mortality in older persons.. Am J Epidemiol.

[59] Mehta KM, Yaffe K, Langa KM, Sands L, Whooley MA (2003). Additive effects of cognitive function and depressive symptoms on mortality in elderly community-living adults.. J Gerontol A Biol Sci Med Sci.

[60] Takeshita J, Masaki K, Ahmed I, Foley DJ, Li YQ (2002). Are depressive symptoms a risk factor for mortality in elderly Japanese American men?: the Honolulu-Asia Aging Study.. Am J Psychiatry.

[61] Fredman L, Magaziner J, Hebel JR, Hawkes W, Zimmerman SI (1999). Depressive symptoms and 6-year mortality among elderly community-dwelling women.. Epidemiology.

[62] Pulska T, Pahkala K, Laippala P, Kivela SL (1997). Six-year survival of depressed elderly Finns: a community study.. Int J Geriatr Psychiatry.

