# Supplementary material for: Depression, Antidepressant Use and Mortality in Later Life: The Health in Men Study
Source: PLoS One. 2010 Jun 23;5(6):e11266. doi: 10.1371/journal.pone.0011266 (PMC2890408; doi:10.1371/journal.pone.0011266)
Supplement: Table S1 — (0.16 MB DOC) [file pone.0011266.s001.doc]

**Table S1.** Summary of observational studies investigating the association between depression in later life and mortality

| Study | Design | Participants | Follow up in years | Ascertainment of depression | Covariates | Findings |
| --- | --- | --- | --- | --- | --- | --- |
| Schoevers et al., 2009 [38] | Cohort | N=3,746  Age 65+ | 10 | GMS-AGECAT (no depression, mild, moderate and severe) | Age, gender, education, marital, myocardial infarction, stroke, cancer, lung disease, diabetes, epilepsy, Parkinson’s disease, arthritis, MMSE, ADL | - 1,844 deaths (all causes) - Crude MHR (mild NS; moderate 1.46, 95%CI=1.18-1.81; severe 1.35, 95%CI= 1.08-1.68) - Fully adjusted models NS - Model adjusted for demographics + morbidities: moderate 1.36, 95%CI= 1.09-1.69; severe 1.41, 95%CI= 1.12-1.77) |
| Surtees et al. 2008 [50] | Cohort | N=8,261 men  N=11,388 women  Age 41-80 | 8.5 | Life Experiences Questionnaire (self-rating – DSM-IV major depression, past 12 months & lifetime) | Age, sex, smoking, systolic blood pressure, total cholesterol, physical activity, body mass index, diabetes, social class, heavy alcohol use, antidepressant use | - 274 deaths by ischemic heart disease - Crude MHR men: 3.30, 95%CI=1.82-5.97 - Crude MHR women: 2.02, 95%CI=0.98-4.17   Adjusted MHR for current depression:   - Men: 3.07, 95%CI=1.55-6.08 - Women: 2.05, 95%CI=0.80-5.29   MHR for past depression: NS |
| Ryan et al., 2008 [25] | Cohort | N=7,363  Age 65+ | 4 | CES-D and MINI for DSM-IV major depression  Mild:  16≤CES-D<23  Severe:  DSM-IV or CES-D≥23 | Centre, education, living status, cognitive impairment, high alcohol, smoking, disability, recent hospitalisation, comorbidity (cardiovascular disease, high BP, high cholesterol, diabetes , thyroid problems or cancer in the past 2 y), underweight and obesity | - 165 deaths - No association between depression and mortality in women (except for severe depression and no antidepressants: adjusted MHR: 1.8; 1.1, 2.8). - Men:   Adjusted MHR for mild depression + antidepressants 2.8; 95%CI=1.0-7.7; severe depression + no antidepressants 1.8, 95%CI=1.0-3.3; severe depression + antidepressants 5.3, 95%CI=2.7-10.5) |
| Ahto et al., 2007 [51] | Cohort | N=660  Age 64+  Excluded people with prevalent CHD | 12 | Zung Sefl-Rating Depression Scale≥ 45 | Age, marital status, social status, n medicines | - 250 deaths - Adjusted MHR for men 1.05, 95%CI=1.00-1.10 - Adjusted MHR for women 1.09, 95%CI=1.03-1.16   Includes information on causes of death |
| Gallo et al., 2007 [52] | RCT | N=1,226 patients from 20 practices  Age 60+ | 5 | Major Depression (DSM-IV) based on SCID-I | Intervention vs usual care group  Analyses adjusted for intervention practice, depression by intervention interaction, age, gender education smoking, cardiovascular disease, stroke, diabetes, cancer, MMSE, suicidal ideation | - 223 deaths - Adjusted MHR=1.65, 95%CI=1.20-2.26   Includes information on causes of death |
| Ben-Ezra et al., 2006 [53] | Panel data | N=1,369  Age 75-94 | 12 | CES-D≥16 | None | - 1,185 deaths - Crude MHR= 0.92, 95%CI=0.80-1.18 |
| Adamson et al., 2005 [54] | RCT  Participants selected if they were randomly allocated to the ‘universal arm’, which included assessment by a nurse | N=13,097  Age 74+ | 3 | GDS-15≥6 | Age, sex, socioeconomic, chronic illness, disability, cognition, drinking and smoking | - 2,804 deaths - Adjusted MHR=1.27, 95%CI=1.11-1.45 |
| van den Brink et al., 2005 [55] | Selected Cohort (with available data)  (3 ≠ countries) | N=1,141 men  Age 70+ | 10 | Zung scores (lowest tertile used as reference) | Self-rated health, chronic diseases, and disability | - 168 deaths - Adjusted MHR=1.17, 95%CI=0.92-1.44 and 1.42, 95%CI=1.14-1.77 for middle and highest tertile of depressive scores. |
| Wulsin et al., 2005 [56] | Cohort | N=3,634  Age 30-91 | 6 | CES-D≥16 | Sex, age, smoking, hypertension, diabetes, BMI, cholesterol, alcohol use | - 127 deaths - CES-D≥16 not associated with mortality MHR=1.50, 95%CI=0.93-2.44 - Second tertile: MHR=1.33, 95%CI=0.86-2.04 - Third tertile: MHR=1.88, 95%CI=1.22-2.91 |
| Blazer et al., 2004 [57] | Cohort | N=4,162  55% Afro-Americans  Age 65-105 | 10 | CES-D  Scale divided into negative, positive, somatic and interpersonal subscores | Crude  Age, sex, race marital status, education, income, cognitive impairment, functional impairment and positive affect. | - 2123 deaths - Crude OR=1.08, 95%CI=1.05-1.10 associated with negative affect - NS on multivariate Cox regression |
| Wilson et al., 2003 [58] | Cohort | N=851  Mean Religious Order Study  Mean age 75.4 | 4.7 | CES-D | Cognitive function, lower limb function, number of chronic illnesses, number of alcohol dirnks, hx smoking, BMI | - 164 deaths      - Adjusted MHR=1.13, 95%CI=1.04-1.24 |
| Mehta et al., 2003 [59] | Cohort  AHEAD Study | N=6,301  70+ | 2 | CES-D8 tertiles  Cognition: MMSE + TICS | Age, gender, education, net worth, marital, race, comorbid conditions, current smoking, lowest tertile of BMI, dependence in ADLs | - 548 deaths   Worst tertile of CES-D8 scores compared with score 0   - MHR=2.1, 95%CI=1.3-3.4 – best cognition - MHR=2.6, 95%CI=1.7-4.1 – medium cognition - MHR=3.1, 95%CI=2.0-4.7 – worst cognition |
| Takeshita et al., 2002 [60] | Cohort (Honolulu) | N=3,196  Age 71-93 | 6 | CES-D (short)≥9 | Age, marital status, antidepressant use | - 684 deaths - Adjusted MHR=1.27, 95%CI=1.01-1.60   (no longer significant after inclusion of chronic diseases in the model) |
| Ensinck et al., 2002 [5] | Retrospective cohort based in general practice | N=68,965  Age 20+  Mean age: 43.9 for depressed and 37.8 for non-depressed | 10-25  average 15 | Diagnosis as recorded in files: depressive disorder or affective psychosis | Age, sex, socioeconomic status | - 4,299 deaths - Adjusted MHR=1.39, 95%CI=1.16-1.65 - NS if onset before age 50 (MHR=1.15, 95%CI=0.79-1.67); after age 50 MHR=1.46, 95%CI=1.20-1.77 |
| Schoevers et al., 2000 [8] | Cohort | N=4,501  AMSTEL  Age 65-84 | 6 | GMS-AGECAT  Neurotic and psychotic depression | Age, education, marital, MI, stroke, other diseases, MMSE, ADL, IADL, disability | - 496 men and 539 women died. - MRR=0.99, 95%CI=0.79-1.23 for women - MRR=1.60, 95%CI=1.20-2.15 for men |
| Fredman et al., 1999 [61] | Cohort | N=764 white women  Age 65+ | 6 | CES-D≥16 |  | - 764 deaths - MHR=1.67, 95%CI=1.15-2.43, adjusted for age - Adjustment for number of medical conditions: scores 2-15 and 25+ associated with greater mortality hazard in people with at least one medical condition (reference, score 0 or 1) |
| Penninx et al., 1999 [6] | Cohort | N=3,056  Age 55-85 | 4 | CES-D≥16  DSM-IV major depression  (CES-D≥16 and not DSM-IV=minor depression) | Age, education, urban, chronic diseases, physical disability, smoking, body mass index, physical activity | - 561 deaths - MRR=1.68, 95%CI=1.00-2.84 for major depression - MRR=1.45, 95%CI=1.08-1.95 for men with minor depression - MRR=0.92, 95%CI=0.65-1.34 for women with minor depression   Includes information on causes of death |
| Zheng et al., 1997 [7] | Cohort  Administrative data | N=57,897  Age 25+ | 2.5 | Self-reported diagnosis of ‘major depression’ and ‘physician diagnosed’ | Age, education, marital status and BMI | - 1,499 deaths - Men: Adjusted MHR=2.4, 95%CI=1.4-4.2 for physician diagnosis - Women: Adjusted MHR=1.0, 95%CI=0.4-2.6 for physician diagnosis - Men: Adjusted MHR=2.6, 95%CI=1.6-4.3 for self-report - Women: Adjusted MHR=1.6, 95%CI=0.8-3.2 for self-report   Includes information on causes of death |
| Pulska et al., 1997 [62] | Cohort | N=1,272  Age 60+ | 6 | Zung Self-Rating Depression Scale  +  DSM-III criteria | Age, sex, marital, education, smoking | - N deaths not reported - MRR=1.3, 95%CI=0.98-1.65 |

N= number of participants; MHR= Mortality Hazard Ratio; MRR= Mortality Relative Risk; NS= not significant; CES-D= Center for Epidemiological Studies Depression Scale; GDS= Geriatric Depression Scale; GMS-AGECAT= Geriatric Mental State Interview and Diagnosis; MMSE=Mini-Mental State Examination; TICS=Telephone Interview for Cognitive Status.
